# Efficacy and Safety of Remdesivir over Two Waves of the SARS-CoV-2 Pandemic

**DOI:** 10.3390/antibiotics10121477

**Published:** 2021-12-01

**Authors:** Mariacristina Poliseno, Crescenzio Gallo, Donatella Concetta Cibelli, Graziano Antonio Minafra, Irene Francesca Bottalico, Serena Rita Bruno, Maria Luca D’Errico, Laura Montemurro, Marianna Rizzo, Lucia Barbera, Giacomo Emanuele Custodero, Antonella La Marca, Donatella Lo Muzio, Anna Miucci, Teresa Antonia Santantonio, Sergio Lo Caputo

**Affiliations:** 1 Unit of Infectious Diseases, Department of Clinical and Experimental Medicine, University of Foggia, 71122 Foggia, Italy; donatellacibelli@yahoo.it (D.C.C.); grazianominafra@gmail.com (G.A.M.); ibott4@gmail.com (I.F.B.); serenaritabruno@gmail.com (S.R.B.); m.derrico1990@gmail.com (M.L.D.); laura.monty91@gmail.com (L.M.); mariannarizzo_@libero.it (M.R.); lucia.barbera@outlook.it (L.B.); g.custodero88@gmail.com (G.E.C.); antonella89la.marca@gmail.com (A.L.M.); dony79@gmail.com (D.L.M.); annamiucci91@gmail.com (A.M.); teresa.santantonio@unifg.it (T.A.S.); sergiolocaputo@gmail.com (S.L.C.); 2 Department of Clinical and Experimental Medicine, University of Foggia, 71122 Foggia, Italy; crescenzio.gallo@unifg.it

**Keywords:** remdesivir, COVID-19 waves, real life-study

## Abstract

The aim of this study is to describe the features, the outcomes, and the clinical issues related to Remdesivir administration of a cohort of 220 patients (pts) with COVID-19 hospitalized throughout the last two pandemic waves in Italy. One hundred and nine pts were enrolled from 1 September 2020, to 28 February 2021 (Group A) and 111 from 1 March to 30 September 2021 (Group B). Notably, no differences were reported between the two groups neither in the timing of hospitalization. nor in the timing of Remdesivir administration from symptoms onset. Remarkably, a higher proportion of pts with severe COVID-19 was observed in Group B (25% vs. 10%, *p* < 0.001). At univariate and multivariate analysis, rather than the timing of Remdesivir administration, age, presence of coexisting conditions, D-dimers, and O2 flow at admission correlated positively to progression to non-invasive ventilation, especially for patients in Group B. However, the rate of admission in the Intensive Care Unit and/or death was comparable in the two groups (7% vs. 4%). Negligible variations in serum GOT, GPT, GGT, and eGFR levels were detected. A mean reduction in heart rate was noticed within the first three days of antiviral treatment (*p* < 0.001). Low rate of ICU admission, high rate of clinical recovery, and good drug safety were observed in COVID-19 patients treated with Remdesivir during two diverse pandemic waves.

## 1. Introduction

Almost two years after its outbreak in Wuhan in December 2019, the current epidemiological scenario of the Severe Acute Respiratory Coronavirus 2 (SARS-CoV-2) pandemic is still serious and is constantly evolving [1].

To date, out of a total population of 60,244,639 [2], in Italy, the SARS-CoV-2 epidemic recorded a total number of 4,774,783 cases and 132,120 deaths according to the latest update of the ISS [3].

The distribution of Coronavirus Disease-2019 (COVID-19) cases in Italy over the year 2020 was heterogeneous and significantly influenced by the adoption of collective and individual restrictive measures which, at first, repressed the significant increase of COVID-19 cases observed at the beginning of 2020 (first “wave”).

**Figure 1:**
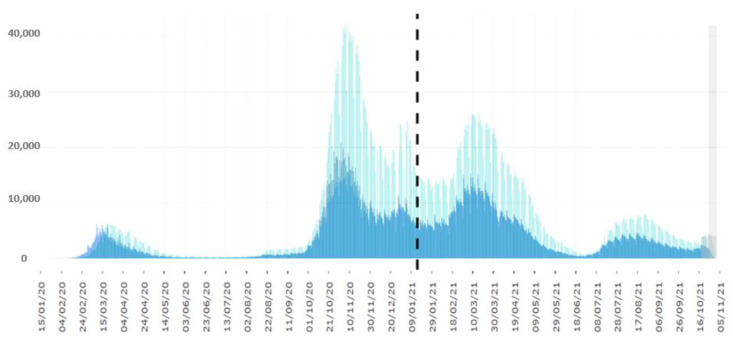
Distribution of COVID-19 cases in Italy between the years 2020 and 2021 (separed by the dotted line). Data are pdated at 2 November 2021 [4].

After a partial relief in summer, the second, significant “wave” of viral diffusion was reported in October 2020, followed by a slight decrease until February 2021, as reported in Figure 1.

By the end of the year 2020, the availability of vaccines, along with the effect of regional public health preventive measures, have contributed to curbing viral diffusion, smoothing the peak of COVID-19 cases from March to June 2021.

In the meantime, SARS-CoV-2 has continued to change, creating new variants with the potential to affect the transmission, disease severity, diagnostics, therapeutics, and natural and vaccine-induced immunity.

As to 8 November 2021, data reported by the Integrated Surveillance System of ISS from genotypic sequencing performed on 72,064 SARS-CoV-2 positive specimens collected since 28 December, outlined the rise of the predominance of the B.1.617.2 Variant of Concern (VOC) and relative under-lineages. Notably, the VOC B.1.617.2, which has accounted for the 45.1% of all specimens sequenced during the year 2020, has been detected in the 91.2% of the specimens collected in the 45 day period from 1 October to 8 November 2021 [5].

Consequently, while the vaccination campaign in Italy is slowly being completed, approximately 2000 new confirmed cases of SARS-CoV-2 infection and 100 COVID-19 cases requiring hospitalization are still reported daily in the country [3,4].

Besides vaccination, which is capable of reducingthe diffusion of SARS-CoV-2 infection and decreasing its morbidity and mortality [6], disposing of effective treatments to manage the earliest phases of COVID-19 among those who get infected could be the winning strategy to use in the next months of the pandemic.

Remdesivir, a prodrug of an adenosine nucleotide analogue, is an antiviral agent with in vitro broad-spectrum activity against diverse viruses families [7,8,9,10,11]. Thanks to its good antiviral activity and its overall safety demonstrated against Ebola virus disease in the wake of the 2014–2016 Ebola outbreak in West Africa [9,10], Remdesivir was initially considered by the scientific community as a promising “antiviral hope” [12,13].

Today, evidence from clinical trials demonstrate its efficacy in shortening the time to recovery from COVID-19 [14,15,16,17]. Moreover, several in vitro studies [18] have assessed Remdesivir efficacy in new SARS-CoV-2 variants, highlighting a comparable drug efficacy between early SARS-CoV-2 and variants B.1.1.7 and B.1.351, possibly due to the high conservation of nsp12 region [19].

Based on this evidence, Remdesivir has been approved by the FDA for the treatment of SARS-CoV-2 pneumonia, onthe condition of being prescribed in patients with recent infection (inferior to ten days) and low-flow oxygen requirement [20].

Nevertheless, Remdesivir properties of reducing the progression of COVID-19 are still debated [21].

Moreover, the choice of whether or not nominate a patient for antiviral treatment could be difficult in real-life settings, for several reasons.

Among them, unreliable time of symptoms onset obtained from patients’ clinical history; prolonged time interval from patients’ symptoms onset to hospitalization (which can be worsened by the lack of hospital bed places in emergency conditions); a growing number of infections in vaccinated patients not eligible to in-hospital treatment with monoclonal antibodies due to positive serum anti-spike IgG immunoglobulins;technical times required for antiviral drug supply, that occasionally can be exceeded by a rapid and fatal COVID-19 progression.

Up until nowadays, limited data exist regarding the use of Remdesivir in real life [22,23,24,25,26]. Could Remdesivir represent a therapeutic option in the current epidemiological scenario, considering the scarcity of valid therapeutic alternatives available and the frequent, rapid evolution of SARS-CoV-2 pneumonia also in patients with mild COVID-19 and recent symptom onset?

The primary aim of this study is to describe the features, the outcomes, and the clinical issues related to antiviral administration in a cohort of patients with COVID-19 treated with Remdesivir.

The secondary aim is to outline possible variations in the efficacy and safety of the antiviral treatment among patients over the last two pandemic waves in Italy, considering the changes in the virological and epidemiological setting of SARS-CoV-2 infection observed in the country.

## 2. Results

### 2.1. General Characteristics of Study Population

A total of 220 patients, mainly males (139 pts, 63%), mean (SD) age 60 (46–74) years, were enrolled in the retrospective analysis. 

General features of all patients treated with Remdesivir are reported in Table 1. 

At least one cardio metabolic co existing condition was present in 150 subjects (68%), especially hypertension (108 pts, 49%). A slight increase of inflammation markers at admission was reported (median (IQR) C Reactive Protein, CRp = 48 (21–108) mg/dL, median (IQR) Interleukin-6, IL-6 = 17 (7–37) ng/mL, median [IQR] D-dimer = 755 (453–1409)) ng/mL). 

Remdesivir was prescribed after a median (IQR) 7 (4–9) days from symptoms onset. Notably, 25 pts (11%) were already receiving oxygen support in High Flow Nasal Cannulas (HFNC) and 8 pts (4%) in non-invasive ventilation (NIV) at the moment of Remdesivir administration.

After a median (IQR) 15 (11–23) days, clinical recovery was observed in 197 pts (89%), 159 of whom also reported virological recovery after a median (IQR) 20 (12–28) days.

Then, the total population was divided into two distinct groups according to the date of hospital admission (see *Methods* section):

- Group A: 109 patients admitted from 1 September 2020 to 28 February 2021;
- Group B: 111 patients admitted from 1 March to 30 September 2021.

As shown in Table 2, no significant differences were observed between Group A and B in regard to the time of hospitalization from symptom onset (median (IQR) 5 (3–8) vs. 6 (3–8) days respectively (*p* = 0.134). The timing of Remdesivir administration from symptom onset was also comparable: median 7(5–9) in Group A vs. 7 (4–9) days in Group B respectively (*p* = 0.453). 

Age, gender, the prevalence of coexisting cardio metabolic conditions, and baseline inflammation markers at admission were also similar in the two groups. Remarkably, a higher proportion of pts with severe COVID-19 (56 pts, 25% vs. 23 pts, 10%, *p* < 0.001) and a higher proportion of patients evolving from low flow oxygen support to NIV (29 pts, 13% vs. 15 pts, 7%, *p* = 0.028) was observed in Group B compared to Group A.

Nevertheless, the rate of patients dead or admitted in ICU were comparable between patients in Group A and Group B (15 pts, 7% vs. 9 pts, 4%, *p* = 0.200).

Differences reported in median (IQR) duration of hospital stay (16 (11–25) vs. 15 (10–22) days, *p* = 0.412) and median (IQR) time to first negative SARS-CoV-2 PCR on nasal-pharyngeal swab (21 (13–35) vs. 19 (12–24) days, *p* = 0.062) was also non-significant between the two Groups.

### 2.2. Remdesivir Efficacy

Correlation tests performed for the whole population treated with Remdesivir highlighted a significant positive correlation between date of hospitalization (r = 0.219, *p* = 0.001), age (r = 0.159, *p* = 0.020), presence of coexisting conditions (r = 0.199, *p* = 0.003), elevated baseline inflammation markers [D-dimer (r = 0.195, *p* = 0.004, C Reactive Protein (CRP) r = 0.186, *p* = 0.006, Inter-leukin 6 (IL-6) r = 0.172, *p* = 0.014)] high-flow oxygen support required at admission (r = 0.423, *p* < 0.001) and progression to non invasive ventilation. 

Conversely, a significant negative correlation was outlined between clinical recovery and age (r = −0.192, *p* = 0.005), presence of coexisting conditions (r = −0.233, *p* = 0.001), elevated D-dimer (r = −0.238, *p* < 0.0001) and IL-6 (r = −0.157, *p* = 0.025) at admission and high-flow oxygen support required at baseline (r = −0.268, *p* = 0.001).

Other correlations are reported in Table 3. Notably, no correlation was observed between the time of Remdesivir administration from symptom onset and neither of the variables selected. 

A naïve Bayesian Classifier (see *Methods* section) was modeled for each group of patients.

**Figure 2:**
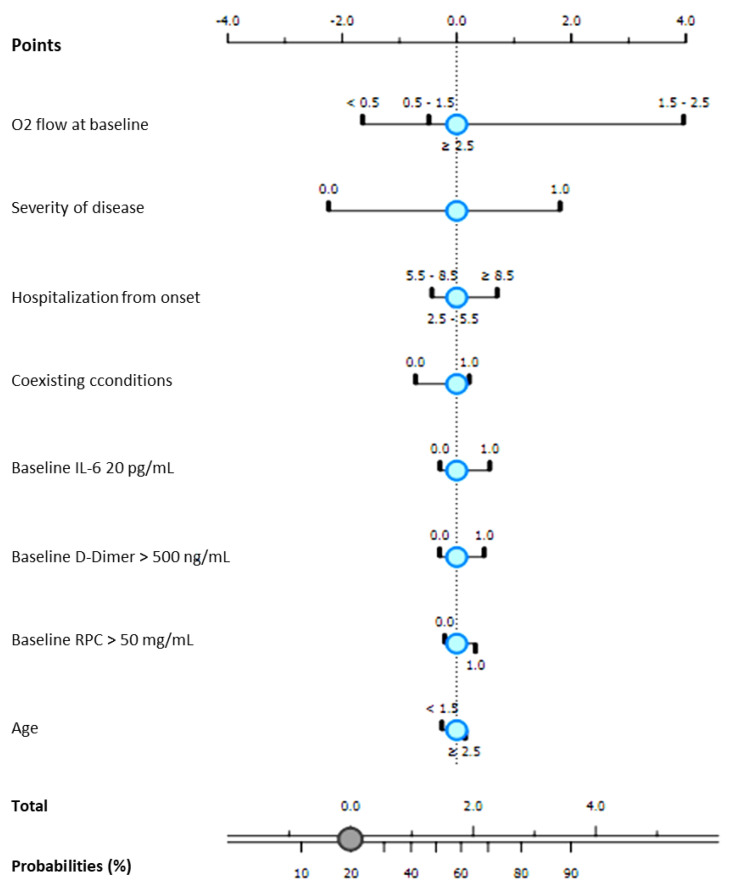
Probability of progression to non invasive ventilation according to Bayesian classifier (NBC). Patients treated with Remdesivir hospitalized during the second COVID-19 wave (September 2020–February 2021—Group A).

**Figure 3:**
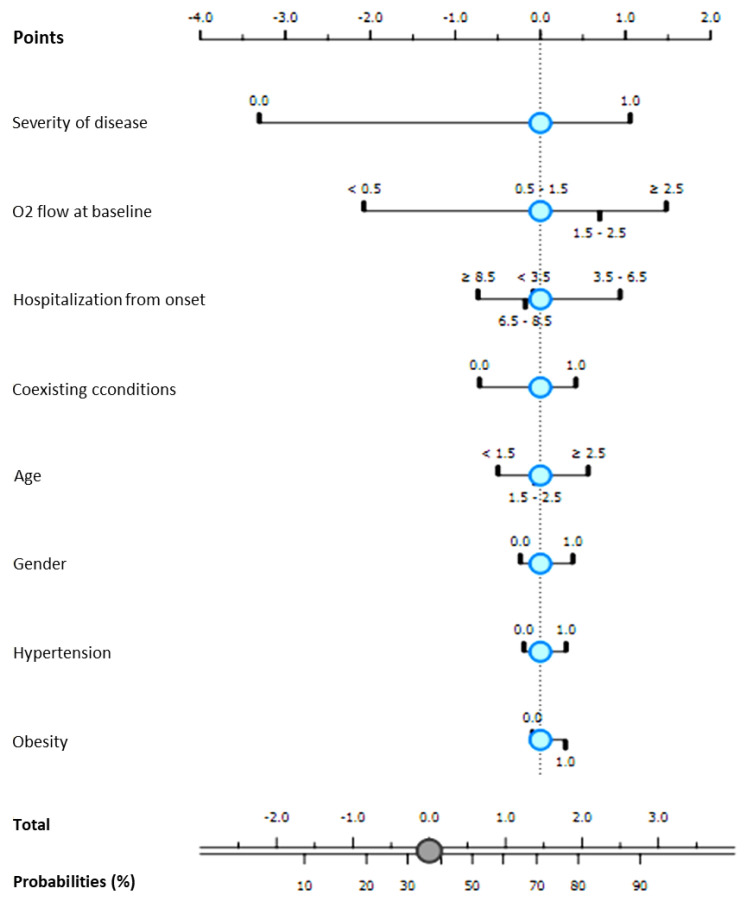
Probability of progression to non-invasive ventilation according to Bayesian classifier (NBC). Patients treated with Remdesivir hospitalized during the second COVID-19 wave (March 2021–September 2021—Group B).

For both groups, all receiving Remdesivir in association to standard of care, the independent probability of progression to NIV in accordance to first eight best-ranked variables identified at univariate analysis among patients’ clinical and laboratory findings admission, was modeled. Results are graphically reported in Figure 2 and Figure 3.

For Group A, the NBC showed that classification accuracy (CA) reached 90.8% and Area Under Curve (AUC) 0.966 considering patients’ O_2_ flow required at baseline, severity of disease, time of hospitalization from symptom onset and inflammation markers at admission, modeling a 20% risk of progressing to NIV.

For Group B, a CA 86% and AUC 0.917 were obtained considering patients’ severity of disease, O_2_ flow at baseline, time of hospitalization from symptom onset, age, gender, and coexisting condition, in particular hypertension and obesity, predicting a 40% risk of progressing to NIV. 

The time of Remdesivir administration was not considered in both models as not significant.

### 2.3. Remdesivir Safety

#### 2.3.1. Liver and Kidney Toxicity

The analysis included 205 patients with at least one Glutamic Oxaloacetic Transaminase (GOT), Glutamin Piruvic Transaminase (GPT), Gamma Glutammil Transpeptidase (GGT), and esteemed Glomerular Filtration Rate (Egfr) assessment available at baseline and within seven days from Remdesivir suspension.

The median (IQR) baseline GOT value detected was 28 (21–40) UI/mL; a decrease to a median (IQR) value of 22 (17–31) UI/mL (*p* < 0.001) was observed by the end of the treatment. 

Conversely, a slight, non-significant, increase in GGT from a median (IQR) value of 37 (23–61) to 44 (26–74) UI/mL (*p* = 0.101) was observed, along with a significant increase (*p* < 0.001) of GPT serum levels from a median (IQR) baseline value before Remdesivir of 28 (19–40) to a final median value of 37 (22–61) UI/mL after Remdesivir. Notably, the analysis was repeated on a subset of patients (19 subjects) with altered baseline GPT values (≥2 but ≤5 times the normal value): mean (SD) baseline value was 112 (33) UI/mL vs. 106 (57) UI/mL reported after Remdesivir administration (*p* = 0.590).

The median (IQR) baseline eGFR was 91 (72–107) mL/min, which slightly increased to a median (IQR) of 100 (83–114) mL/min (*p* < 0.001). Furthermore, in this case, the analysis was repeated on a subset of 28 subjects in which Remdesivir was administered with eGFR values >30 mL/min but <60 mL/min. Mean reported baseline value was 47 mL/min; mean reported eGFR value after Remdesivir was 65 mL/min (*p* = 0.002).

#### 2.3.2. Heart Safety and Cardiac Rhythm Abnormalities

There were 133 patients included in the study of Remdesivir heart safety, as they underwent heart rhythm monitoring through serial Electrocardiograms (ECGs) (see *Methods* section). They were mainly males (83 pts, 63%), mean age 60 (57–73) years, and already presented cardiovascular comorbidities, such as chronic atrial fibrillation (17 pts, 32%), history of acute myocardial infarction (14 pts, 11%), history of chronic heart disease (14 pts, 11%). Notably, 55 pts (42%) presented with severe COVID-19. 

**Figure 4:**
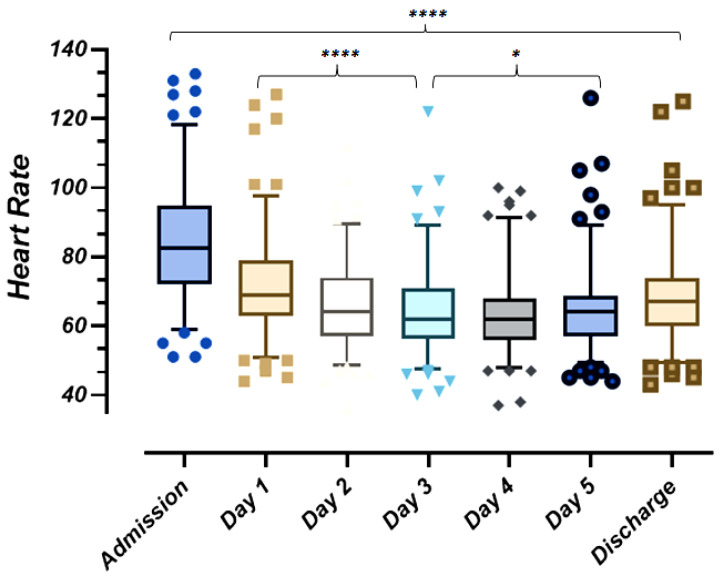
Box plot showing heart rate changes in course of Remdesivir administration on a subset of 133 patients.* *p* < 0.05; **** *p* < 0.001.

Compared to baseline, over a median (IQR) of 6 (6–7) ECGs performed in the course of hospitalization, a significant reduction of heart rate to a minimum of 36 bpm was observed after administration of Remdesivir (Kruskal-Wallis *p* < 0.0001) from mean (SD) 84 (17) bpm at admission to 62 (13) bpm at discharge. The heart rate reduction was more significant from day 1 to day 3 of antiviral administration (Kruskal-Wallis *p* < 0.001) than from day 3 to day 5 (Kruskal-Wallis *p* < 0.05), as shown in Figure 4.

The median (IQR) heart rate reduction observed was 22 (14–34) bpm. Heart rate reduction was proportional to baseline heart rate values (r = 0.784, *p* < 0.001), age (r = −0.230, *p* = 0.008), presence of arterial hypertension (r = −0.203, *p* = 0.02). In 70 pts (53%) prolongation of QTc interval > 440 ms in women and >460 ms in men was reported. New-onset heart rhythm abnormalities were noticed in 13 pts (10%). six cases of atrial fibrillation and seven cases of isolated supraventricular extrasystoles were documented. 

In any case, the occurrence of clinical symptoms related to bradycardia, to other cardiac rhythm abnormalities, or to QTc prolongation implied Remdesivir discontinuation.

## 3. Discussion

Remdesivir is a pro-drug of an adenosine nucleoside triphosphate analog [27]. After being converted in its active form, Remdesivir interferes with the action of viral RNA-dependent RNA polymerase and evades proofreading by viral exoribonuclease (ExoN), causing a decrease in viral RNA production [28,29].

In some viruses, such as the respiratory syncytial virus, Remdesivir causes the RNA-dependent RNA polymerases to pause, but its predominant effect (as in Ebola) is to induce an irreversible chain termination. Unlike with many other chain terminators, this is not mediated by preventing the addition of the immediately subsequent nucleotide but is instead delayed, occurring after five additional bases have been added to the growing RNA chain [30]. Hence, Remdesivir is classified as a direct-acting antiviral agent that works as a delayed chain terminator.

Data from worldwide clinical trials have concluded that the five-day course treatment with Remdesivir is effective in reducing time to recovery and duration of hospital stay in patients with moderate to severe SARS-CoV-2 pneumonia requiring low flow oxygen support [14,15,16,17].

Moreover, Remdesivir efficacy in reducing both SARS-CoV-2 viral RNA and subgenomic RNA has been demonstrated both in vitro and in vivo [31,32].

Remdesivir is currently approved in Italy to treat patients hospitalized with SARS-CoV-2 pneumonia having recent symptom onset and requiring low-flow oxygen ventilation [20].

Nevertheless, interim results from the Solidarity Trial of 11,330 patients randomized to receive four repurposed antiviral drugs—remdesivir, hydroxychloroquine, lopinavir, and interferon beta-, show no effect of Remdesivir (administered in 2750 subjects) on mortality, initiation of ventilation, or duration of hospital stay, neither in patients with low oxygen requirement [21].

The enrollment of patients with COVID-19 of diverse stages and severity [14], the absence of a standardized method of measure of Remdesivir efficacy [15,16,17], and, in some trials, the absence of a Remdesivir-free control arm [15], could partially explain the controversial results of clinical trials, that should also be re-interpreted on the basis of what we have learned about the complex and rapid evolution of the natural course of COVID-19 from clinical practice.

Recent real-life studies support the use of Remdesivir as related to a reduction in mortality rate compared to placebo [23,25,26] and associated with a good safety profile [23,26].

This is the first report, in Italy, of Remdesivir use in patients hospitalized in the course of the year 2021. 

The scenario of the SARS-CoV-2 pandemic in Italy has enormously changed in the course of the last year, due to the beginning and the progression of the vaccination campaign, the availability of new post-exposure treatments as monoclonal antibodies, and, lastly, because of the emergence of new SARS-CoV-2 variants with recognized increased transmissibility and pathogenicity, enhanced ability to evade detection by diagnostic tests, decreased susceptibility to therapeutic agents (i.e., monoclonal antibodies) and good capacity to escape natural or vaccine-induced immunity [33,34].

In our observation, patients treated with Remdesivir from March to September 2021, were slightly younger and substantially similar in regard to clinical and laboratory features at admission to subjects observed during the first five months of the study.

Fortunately, a negligible proportion of vaccinated subjects with COVID-19 was admitted over the year 2021. Nevertheless, a higher number of severe forms of COVID-19 disease and, consequently, a significant propensity towards a respiratory worsening requiring non invasive ventilation were observed among this group of patients.

It is worth mentioning that patients in both groups were hospitalized and received Remdesivir after approximatively one week from symptom onset. 

Both the difficulties in defining the precise symptom onset from patients stories and the common *wait-and-see-attitude* of General Practitioners contribute to (i) causing a bias in the calculation of the precise time of symptom onset and (ii) producing a delay in the timing of hospital admission from the onset of the infection. The latter condition, in particular, could explain the correlation observed between baseline oxygen flow requirement and the progression towards non-invasive ventilation, which can be also influenced by different timing and doses of steroid treatments prescribed at home, not included in our data collection.

The importance of identifying a rapid strategy to contrast COVID-19 progression, (i.e., post-exposure prophylaxis with monoclonal antibodies or early start of antiviral treatment) is clearly highlighted by our analysis. 

Following what was already reported in the literature [35], our data have confirmed that the presence of pre-existing comorbidities and the elevation of baseline inflammation marker have a great impact on the progression of COVID-19. This data suggest that the careful observation of patients at admission and their frequent re-evaluation in the course of hospitalization is essential for choosing the proper treatment accordingly to individual characteristics and COVID-19 severity and progression.

Nevertheless, despite the higher clinical complexity of patients hospitalized in recent times, in our experience a good recovery rate and a very small proportion of patients admitted in Intensive Care Unit or dead over the total population was observed, suggesting that the antiviral treatment has been an efficacious strategy regardless from the date of patients’ admission.

We investigated Remdesivir cardiac, renal, and hepatic safety.

Due to its real life-design, our study allowed us to observe the effects of a five-day course treatment with Remdesivir in patients with mild renal and hepatic impairment at admission. A negligible variation in glomerular filtration rate and liver enzymes, more related to the evolution of the viral infection than to drug-related toxicity, was reported. Moreover, despite a significant reduction in heart rate observed after Remdesivir administration, no serious cardiovascular toxicity was observed in patients with COVID-19, even in those with severe disease and cardiovascular comorbidities.

Our study has some limitations. 

The first is its monocentric design: as a consequence, results might be affected by local practice in the management of COVID-19. Secondly, its retrospective nature, that did not allow to include all subjects in the analysis of renal and hepatic safety and in the study of heart rate abnormalities, due to the lack of blood samples and ECGs. 

Lastly, we are aware that the absence of a Remdesivir-free control group could affect the completeness and the reliability of the results, which should therefore be interpreted cautiously also because the study was not conducted with randomized groups that could limit the presence of confounding factors. 

Nevertheless, this report provides several insights from clinical practice and tries, for the first time, to define the utility of antiviral treatment in the context of two diverse pandemic waves.

## 4. Materials and Methods

### 4.1. Data Collection

All patients hospitalized in the COVID-19 Infectious Diseases Unit in Foggia University Hospital from September 1, 2020 to September 30, 2021 were retrospectively included in the analysis at the condition of meeting the following criteria: (i) age ≥ 18 years; (ii) diagnosis of SARS-CoV-2 infection confirmed with positive Polymerase Chain Reaction (PCR) on nasal-pharyngeal swab; (iii five-day course treatment with Remdesivir.

After anamnesis and clinical evaluation, Remdesivir was prescribed in patients with recent symptom onset (inferior to ten days), radiological evidence of SARS-CoV-2 pneumonia, and low flow Oxygen requirement, according to international guidelines and the Italian Agency of Drug indications [36,37].

It must be mentioned that, in a limited number of cases, patients requiring oxygen support from HFNCNIV were considered eligible to treatment with Remdesivir upon clinician’s judgement on a case-by-case basis, independently from the purpose of this study. However, the time criterion (symptom onset inferior to ten days) was fulfilled in all cases.

Besides antiviral treatment, all patients received *standard of care* therapy for SARS-CoV-2 pneumonia: Dexamethasone 6 mg once daily, Low Molecular Weight Heparin (LMWH) 80 mg/kg once daily or 100 mg/kg twice daily according to clinical evidence of acute pulmonary embolism and/or specific cardiac conditions, antimicrobial prophylaxis with Ceftriaxone 2 gr or Levofloxacin 750 mg once daily.

Eligibility to Remdesivir was established at admission and the drug was requested upon nominal demand to the Italian Agency of Drugs and delivered by hospital pharmacy within approximatively 48 h. Contraindications included (i) GPT ≥ 5 times the normal range values (40 UI/mL), (ii) eGFR < 30 mL/min, hemodialysis, or peritoneal dialysis, (iii) evidence of severe bradycardia (heart rate < 55 bpm, or <65 bpm in presence of symptoms) at ECG performed at admission.

Once provided, antiviral treatment was started and continued regardless of a potential worsening in clinical conditions.

Conversely, increase in GPT ≥ 5 times the normal range values, decrease of eGFR below 30 mL/min, evidence of symptomatic bradycardia (the association of a heart rate <60 bpm and the development of clinical manifestations of syncope or presyncope, transient dizziness, or light-headedness, heart failure symptoms, or confusedstates), represented reasons for antiviral discontinuation. 

No treatment interruption for any cause was recorded.

All patients received a blood test including dosage of D-dimers, IL-6, CRP, GOT, GPT, GGT serum levels, eGFR at baseline, during and after Remdesivir administration according to clinical necessities. 

All patients had one ECG recorded at admission and discharge.

Starting from February 2021, due to asymptomatic sinus bradycardia during Remdesivir administration in a subset of patients of the same cohort [38], daily ECGs in the course or immediately after Remdesivir administration were scheduled for all patients. 

Data regarding heart rate and related new-onset abnormalities, liver and kidney function monitoring were reported in the case report form.

Demographics, clinical data, and data regarding COVID-19 medical history (vaccination, data of first positive and first negative SARS-CoV-2 PCR, date of hospital admission and discharge, date of Remdesivir administration, co-medications, oxygen support required at admission) were retrieved from medical charts.

The timing of hospitalization from symptom onset and timing of Remdesivir administration from symptoms onset were calculated, along with the duration of hospital length of stay and time to first negative SARS-CoV-2 PCR on a nasal-pharyngeal swab.

The patient’s outcome and severity of disease were established after discharge or transfer to the Intensive Care Unit.

We defined “Clinical recovery” as the stable remission of both symptoms and signs of infection recorded at the patient presentation, along with (i) a stable reduction CRP to a value <10 mg/L and (ii) a peripheral oxygen blood saturation >95% in room air. 

Nasal-pharyngeal swabs were routinely performed after the suspension of Oxygen therapy. At the condition that clinical recovery was achieved, negative SARS-CoV2 PCR on nasal-pharyngeal swabs was preferred, but not mandatory, to allow patients discharge.

We defined “Virological recovery” as the association of clinical recovery and negative SARS-CoV-2 PCR on nasal pharyngeal swab performed before discharge. 

The severity of disease was defined in accordance with current WHO indications [39].

### 4.2. Study Design and Statistical Analysis

**Figure 5:**
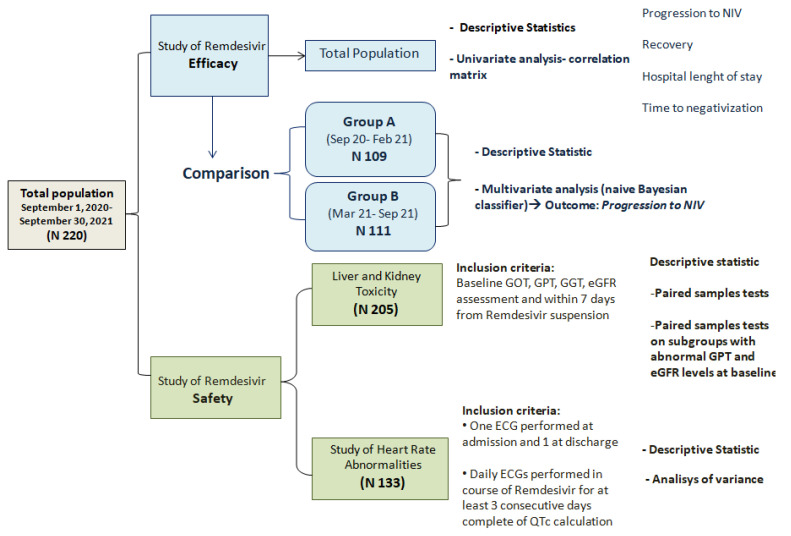
Flow-chart illustrating study design.

Figure 5 shows the design of our research, which was essentially double aimed:

- A primary endpoint was to report on the efficacyof the five days course treatment with Remdesivir.

To this aim, a descriptive statistic of clinical characteristics and outcomes, oxygen support, and laboratory markers at baseline of all patients treated with Remdesivir in our Unit in the study period was performed. Categorical variables are reported in terms of absolute number and percentages; continuous variables are described as means (Standard Deviation, SD) or medians (Inter Quartile Range, IQR, QI to Q3) according to their parametric or non-parametric distributions.

Over the course of the study period, covering all year 2021, a significant change in clinical and epidemiological features of new SARS-CoV-2 infections was observed in Italy, due to the rise in the prevalence of SARS-CoV-2 VOC, the increase in the number of new infections in vaccinated subjects [5] and the availability of new treatments for hospitalized patients with mild/moderate COVID-19 [40].

In light of these considerations, we aimed to outline if variation in the efficacy and the safety of the antiviral treatment were observed in course of the year 2021.

To this aim, patients were split, according to the date of hospitalization into two distinct groups mirroring the two last “pandemic waves” observed in Italian country between the end of the year 2020 and the year 2021:

- Group A: patients admitted from 1 September 2020 to 28 February 2021;
- Group B: patients admitted from 1 March to 30 September 2021.

Descriptive statistics were performed for each group. The Chi-square test or Fisher exact test were performed, as appropriate, to compare proportions between Group A and B. Independent samples Student’s *t*-test or Mann–Whitney U-test were used, as appropriate, to compare mean/median values. A *p* < 0.05 was considered statistically significant.

Univariate Analysis (Pearson’s or Spearman’s coefficient calculation as appropriate) was performed for the total population to highlight possible correlation between: (i) Progression to non-invasive ventilation; (ii) Clinical Recovery; (iii) Hospital length-of-stay; (iv) Time from first positive to first negative nasal-pharyngeal swab and patients laboratory and clinical features at baseline, with special focus on the date of hospitalization, the timing of hospitalization from symptom onset and the timing of Remdesivir administration from symptom onset. A *p* < 0.05 was considered statistically significant. Statistics were performed using Jamovi 1.8.4 package.

Multivariate analysis was performed to identify possible predictors of respiratory worsening and progression to non invasive ventilation. 

Orange 3.30.1 package was used to construct a naïve Bayesian classifier model (NBC). In a Bayes classifier, the learning agent builds a probabilistic model of the imputed attributes and uses that model to predict the classification of a selected outcome. In the case of NBC, the independence assumption is made that the input attributes are conditionally independent of each other given the classification [41].

In this study, the NBC was used to model the probability of NIV requirement in accordance with the eight best-ranked variables identified among patients’ clinical and laboratory findings admission. Two distinct NBCs were modeled with attributes of patients in Group A and patients in Group B.

A “leave-one-out” validation was used to compute the classification accuracy, sensitivity, and specificity of the models, and to assess them through Area Under the ROC curve. Results were graphically reported in two distinct nomograms.

- A secondary endpoint of our work was to describe the safety of Remdesivir in terms of drug-induced liver and kidney toxicity and cardiac rhythm abnormalities.

To this end, all patients with serum GOT, GPT, GGT, and eGFR levels available at baseline and within seven days from Remdesivir suspension were enrolled in the analysis. The paired-samples Student’s *t*-test or Wilcoxon Rank were performed to compare transaminase and glomerular filtration rate before and after Remdesivir administration, according to their parametric and non-parametric distribution of the variables. The tests were repeated on a subset of subjects with GOT and GPT levels at admission ≥2 but ≤5 times the normal range and on a subgroup of patients with eGFR values ≥ 30 mL/min but ≤60 mL/min. 

A *p* < 0.05 was considered statistically significant.

The study of cardiac rhythm was performed on a subset of subjects with at least three ECGs available recorded in course of Remdesivir, besides those performed at admission and discharge. 

For ECG, the corrected duration of QT interval (QTc) was calculated in relation to heart rate according to Bazett’s formula [42]. QTc prolongation was defined as the detection of values above the normal range (440 ms for men, 460 ms for women) [43]).Possible presentation of cardiac arrhythmias related to bradycardia, including sinus pause, sinus node arrest, tachycardia-bradycardia, atrioventricular block, atrial flutter, atrial fibrillation [44], and their possible associated clinical complication were also recorded.

Descriptive statistics were performed. Repeated measures were compared withthe non-parametric ANOVA test. (Kruskal Wallis). A *p* < 0.05 was considered statistically significant. Statistics were performed using Jamovi 1.8.4. and GraphPad Prism 9 packages.

## 5. Conclusions

Low rate of ICU admission and/or death and high rate of clinical recovery were observed in our cohort of COVID-19 patients treated with Remdesivir throughout the last two pandemic waves reported in Italy. Notably, higher severity of disease and enhanced probability of progression to non-invasive ventilation was observed in patients hospitalized from March to September 2021. Nevertheless, favorable outcomes were observed in a high proportion of subjects, independently from the date of hospitalization.

Further studies are needed to assess the real advantages of the use of Remdesivir in the current epidemiological scenario and understand to which extent antiviral treatments could be properly used to optimize the management of patients with COVID-19. 

## Figures and Tables

**Table 1:** General features of all patients treated with Remdesivir.

Variables	Overall (N = 220)
**Mean (±SD) age, years**	60 (46–74)
**Gender, n (%)**	
Males Females	139 (63) 81 (37)
**Vaccinated, n (%)**	9 (4)
**Coexisting dismetabolic conditions, n (%)**	
Hypertension Obesity Type II diabetes	102 (49) 54 (26) 53 (25)
**Two or more coexisting conditions, n (%)**	70 (39)
**Median time (IQR) from symptom onset to Hospitalization, days**	6 (3–8)
**Median time (IQR) from symptom onset to Remdesivir, days**	7 (4–9)
**Laboratory tests at admission, median (IQR)**	
RPC, mg/dL IL-6, pg/mL D-dimer, ng/mL GOT, UI/mL GPT, UI/mL GGT, UI/mL eGFR, mL/min	48 (21–108) 17 (7–37) 755 (453–1409) 28 (21–40) 28 (19–40) 37 (23–61) 91 (72–107)
**Oxygen flow required at admission, n (%)**	
Low flow oxygen HFNC NIV	187 (85) 25 (11) 8 (4)
**COVID-19 Severity of Disease, n (%)**	
Mild Moderate Severe Critical	15 (7) 108 (49) 79 (35) 18 (8)
**Progression to Non Invasive Ventilation, n (%)**	60 (27)
**Outcome, n (%)**	
Intensive Caare Unit Admission/death Clinical Recovery	23 (10) 197 (89)
**Virological Recovery, n (%)**	159 (72)
**Median time (IQR) from first positive to first negative SARS-CoV-2 PCR on nasal-pharyngeal swab, days**	20 (12–28)
**Median (IQR) duration of hospital stay, days**	15 (11–23)

SD: Standard Deviation; IQR: Interquartile Range (Q1 to Q3); CRP: C Reactive Protein; IL-6: Interleukin 6; GOT: Glutamic Oxaloacetic Transaminase; GPT: Glutamic Piruvic Transaminase; GGT: Gamma Glutammil Transpeptidase; eGFR: esteemed Glomerular Filtration Rate.

**Table 2:** General features of patients admitted from 1 September 2020 to 28 February 2021 (Group A) and patients admitted from 1 March to 30 September 2021 (Group B). * A *p* < 0.05 (bold) was considered as statistically significant.

Variables	COVID-19		*p* Value *
	Second Wave Group A (N = 109)	Third Wave Group B (N = 111)	
**Mean (±SD) age, years**	62 (50–74)	58 (42–74)	0.057
**Gender, n (%)**			
Males	70 (32)	69 (31)	0.781
Females	39 (18)	42 (19)	
**Vaccinated, n (%)**	0 (0)	9 (8)	**0.003**
**Coexisting cardio metabolic conditions, n (%)**			
Hypertension Obesity Type II diabetes	60 (29) 26 (12) 29 (14)	42 (20) 28 (13) 24 (11)	**0.004** 1.00 0.341
**Two or more coexisting conditions, n (%)**	38 (34)	32 (28)	0.177
**Median time (IQR) from symptom onset to hospitalization, days**	5 (3–8)	6 (3–8)	0.134
**Median time (IQR) from symptom onset to Remdesivir, days**	7 (5–9)	7 (4–9)	0.453
**Laboratory tests at admission, median (IQR)**			
RPC, mg/dL IL-6, pg/mL D-dimers, ng/mL GOT, UI/mL GPT, UI/mL GGT, UI/mL eGFR, mL/min	36 (22–108) 17 (8–36) 735 (398–1568) 25 (20–36) 28 (20–42) 47 (23–69) 86 (69–100)	52 (21–107) 16 (7–37) 794 (504–1222) 30 (23–42) 28 (19–40) 34 (23–63) 97 (79–111)	0.547 0.458 0.313 **0.014** 0.786 0.097 **0.006**
**Oxygen flow required at admission, n (%)**			
Low flow oxygen	94 (43)	93 (13)	**0.032**
HFNC	12 (5)	13 (6)	
*NIV*	3 (1)	5 (2)	
**COVID-19 Severity of disease, n (%)**			
Mild	8 (4)	7 (3)	**<0.001**
Moderate	68 (31)	40 (18)	
Severe	23 (10)	56 (25)	
Critical	10 (94	8 (4)	
**Progression to Non Invasive Ventilation, n (%)**	15 (14)	29 (26)	**0.028**
**Outcome, n (%)**			
Intensive Care Unit admission/Death	15(7)	9 (4)	0.200
Clinical Recovery	94 (42)	102 (46)	
**Virological Recovery, n (%)**	26 (23)	35 (12)	0.203
**Median time (IQR) from first positive to first negative SARS-CoV-2 PCR on nasal-pharyngeal swab, days**	21 (13–35)	19 (12–24)	0.062
**Median (IQR) duration of hospital stay, days**	16 (11–25)	15 (10–22)	0.412

SD: Standard Deviation; IQR: Interquartile Range (Q1 to Q3); RPC: C Reactive Protein; IL-6: Interleukin 6; GOT: Glutamic Oxaloacetic Transaminase; GPT: Glutamic Piruvic Transaminase; GGT: Gamma Glutammil Transpeptidase; eGFR: esteemed Glomerular Filtration Rate.

**Table 3:** Spearman/Pearson’s correlation coefficient matrix between patient’s demographic, clinical and laboratory features and (i) Progression to non-invasive ventilation (NIV); (ii) Clinical recovery, (iii) Hospital length-of-stay, (iv) Time to negativization of SARS-CoV-2 PCR on nasal-pharyngeal swab. A *p* < 0.05 (bold) was considered as statistically significant.

Variables	Progression to NIV		Clinical Recovery		Hospital Length-of Stay		Time to Negativization	
	r	*p*	r	*p*	r	*p*	r	*p*
**Age**	0.159	**0.020**	−0.192	**0.005**	0.291	**<0.001**	0.125	0.129
**Male gender**	0.062	0.363	0.045	0.505	0.089	0.167	0.085	0.302
**Coexisting conditions**	0.199	**0.003**	−0.233	**0.001**	0.222	**<0.001**	0.034	0.682
**D-dimer at admission**	0.195	**0.004**	−0.238	**<0.001**	0.203	**0.003**	0.091	0.276
**CRP at admission**	0.186	**0.006**	−0.127	0.062	0.117	0.087	0.128	0.123
**IL-6 at admission**	0.172	**0.014**	−0.157	**0.025**	0.141	0.045	0.125	0.141
**Oxygen support at admission**	0.423	**<0.001**	−0.268	**0.001**	0.094	0.066	0.115	0.164
**Time from onset to hospitalization**	−0.010	0.885	0.113	0.094	−0.103	0.126	−0.011	0.893
**Time from onset to Remdesivir**	−0.006	0.930	0.019	0.777	−0.036	0.595	0.163	0.048
**Date of hospitalization**	0.219	**0.001**	0.040	0.560	0.018	0.791	−0.076	0.357

RPC: C Reactive Protein; IL-6: Interleukin 6; NIV: non invasive ventilation.
